# Supplementary material for: Orthotropic Piezoelectricity in 2D Nanocellulose
Source: Sci Rep. 2016 Oct 6;6:34616. doi: 10.1038/srep34616 (PMC5052617; doi:10.1038/srep34616)
Supplement: Supplementary Information [file srep34616-s1.pdf]

Supplementary Material for:

"Orthotropic Piezoelectricity in 2D Nanocellulose"

Y. García[1*], Yasser B. Ruiz-Blanco[2*], Yovani Marrero-Ponce[2,3] and C. M. Sotomayor-Torres[1,4]

[1]Catalan Institute of Nanoscience and Nanotechnology (ICN2), CSIC and The Barcelona Institute of Science and Technology, Campus UAB, Bellaterra, 08193 Barcelona, Spain.

[2]Unit of Computer-Aided Molecular "Biosilico" Discovery and Bioinformatics Research (CAMD-BIR Unit), Faculty of Chemistry-Pharmacy. Universidad Central "Marta Abreu" de Las Villas, 54830 Santa Clara, Cuba.

[3]Universidad San Francisco de Quito (USFQ), Grupo de Medicina Molecular y Traslacional (MeM&T), Colegio de Ciencias de la Salud (COCSA), Escuela de Medicina, Edificio de Especialidades Médicas, Hospital de los Valles, Av. Interoceánica Km 12 ½—Cumbayá, e Instituto de Simulación Computacional (ISC-USFQ), Diego de Robles y vía Interoceánica, Quito 170157, Ecuador.

[4]ICREA—Institució Catalana de Recerca i Estudis Avançats, E-08010 Barcelona, Spain.

[1]Catalan Institute of Nanoscience and Nanotechnology (ICN2), Campus UAB, 08193 Bellaterra, Spain.

[*] Correspondence and requests for materials should be addressed to: Y. G.: yamila.garcia@uv.es or Y.B. R.-B.: yasserrb@uclv.edu.cu.

Contents:

1. Definition of a numerical descriptor for a quantification of the electrostatic forces in directional bonds.

2. Understanding electrical forces in the ubiquitous water dimer.

3. On the electrical nature of HB.

3.1. Regarding the limitations of the dipole model for HB within the bond region.

3.2. About the extension of the dipole model to the comprehension of crystalline piezoelectric effects due to localized single-bonds piezoelectricity.

4. Interpolation method for the definition of piezoelectric coefficients.

1. Definition of a numerical descriptor for a quantification of the electrostatic forces in directional bonds.

Our manuscript is focused on the possibility of electromechanical response in 2D crystals of Iβ-NC. Such response is originated by the effect of an external **E** over the electron density of HB. Consequently the characterization of the susceptibility of these HB to electric fields is a necessary feature that depends on the internal response of the HB´s electron cloud. However, there exists a void of knowledge related to classical approaches, in terms of structural/electrical parameters, that can be useful to characterize the behavior of the interactions within the bond region[1,2]. Probably, this absence of theoretical tools is a consequence of that even today most of the phenomena of interest for Science can be described using existing classical approaches, e.g.

the dipole model, or quantum mechanics approaches e.g. to estimate the energy and the structure of atomic systems.

We here define a numerical descriptor[3] (labelled as 't_a') in order to study the effect of external fields on the electromechanical response of bonded atoms. This descriptor accounts for the deviations of the **E**-field along the bond length relative to the behaviour expected for two positive point charges immersed in an uniform electron density region.

We investigate the dependence between the interatomic position (d) and the angle between **E** and the interatomic axis (Φ) , i.e. $\Phi = \Phi(d)$. For ideal behaviours, such dependence is represented by a Heaviside transition between 0° and 180°, in the region between the two extreme atom positions,

$$\Phi_{ideal}(d) = \lim_{t \to 0} \left[\frac{\pi}{2} + \tan^{-1}\left(\frac{(d - d_0)/a_0}{t/a_0} \right) \right] \quad \text{(S1)}$$

In the expression above a_0 is the Bohr radius and d_0 represents the point where the 0-180° step transition takes place. In our context, such d_0-point correspond to the location where **E** changes the orientation relative to the two-atom bond axis. We define the dimensionless descriptor as $t_a = \left| \frac{t}{a_0} \right|$ for finite t - valued functions. This reasoning gives us the following expression for t_a ,

$$t_a = \left| \frac{(d - d_0)/a_0}{\tan\left[\Phi(d) - \frac{\pi}{2} \right]} \right| \quad \text{(S2)}$$

We reported t_a values after statistical treatment for a set of N points $(d, \Phi(d))$ in each of the studied interatomic interactions. The statistical treatment comprises the fitting of such points to

modulated Heaviside functions, eq. S2. The final t_a values, were obtained as the average over nearly a thousand points and the standard deviations for each bond was lower than 10^{-5}.

The behaviour of the **E**-vector along the bond axis is indicative of the electrical feasibility of the chemical bonds. In the case of Iβ-nanocellulose (NC) system we restricted the representation of $\Phi \equiv \Phi(d)$ dependence for three representative hydrogen bonds (HB) pairs and three selected covalent bonds (CB). Deviations from the ideal behaviour are addressed to smooth transitions on the electron density between the pair atoms. The implications of such deviations are highlighted in the main text.

2. Understanding electrical forces in the ubiquitous water dimer

The water dimer offers a feasible and comprehensive frame to study HB[4-6]. Actually, 2●(H_2O) is a manageable system to understand the impact of numerical approximations on final numerical outcomes and to rationalize the response of HB relative to coexistent polar CB.

a

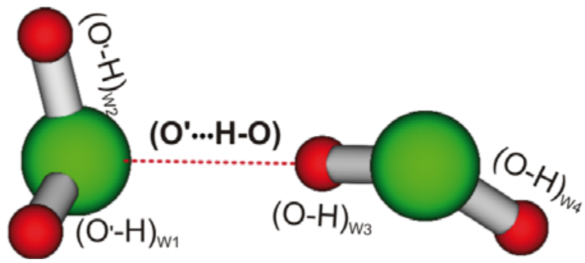

b

Bond Label	Bond Distance (Å)
(O'•••H-O)	1.90
$(O'\text{-}H)_{W1}$	0.96
$(O'\text{-}H)_{W2}$	0.96
$(O\text{-}H)_{W3}$	0.97
$(O\text{-}H)_{W4}$	0.96

Figure S1. Schematic representation of the water dimer. a, By green and red colours we represent oxygen and hydrogen atoms respectively. HB pattern is represented by red dashed lines. **b**, Geometric distances for all the bonds are included in the table.

Fig. S1 includes the scheme of the geometry we use to perform numerical calculations. The structure is the configuration we obtain after fully relaxing the system with 6-31g (d,p) basis set

and using an hybrid functional (HYB-xc) to introduce many-body electron-electron interactions within a reduced mean-field model[7-10]. The HYB-xc is modelled following the methodology already developed by some of us[11]. Here, HYB-xc is defined as 80% PW91[8] and 20% Fock for exchange contributions.

Fig. S2 is indicative of the behaviour of the **E**- field along the axis defined by the HB (O···H) in the water dimer. In a similar trend, Fig. S3 reports **E**-profile of the covalent bond $(OH)_{W1}$ (see Fig.1 for spatial representation of the bonds). The CB depicts a larger gradient in the variation of **E**.

The angle between **E** vector and HB axis is named Φ. The behaviour of the function $\Phi \equiv \Phi(d)$ is represented in the lower panels in Figure´s S2 and S3. It resembles the profile of two positive

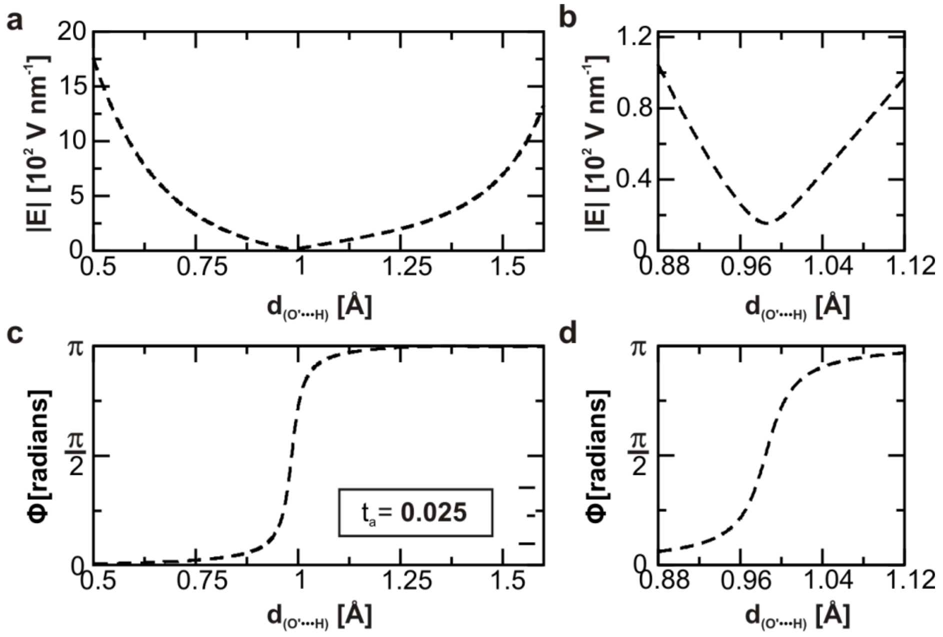

Figure S2. Electrical characterization of HBs for the water dimer. a, Representation of the variation of the electric field modulus (|**E**|) in the two atoms axis. The asymptotic behaviour coincides with O and H positions (positive charge centres in our model). **b**, Characterization of the evolution of the angle between **E** and the two

atom axis, Φ= Φ(d). The deviations of such curves from the ideal Heaviside function are an indication of the electric susceptibility of chemical bonds. The descriptor value included in the inset accounts for this information.

poles instead of a dipole model. This argument supports the claim in favour of a modified electrostatic picture for chemical bonds[12]. The use of the index introduced in (Supp. Mat. S1) for the description of $\Phi \equiv \Phi\left(d\right)$ facilitates the understanding of such electrostatic features and establishes a frame to quantify the electrical feasibility of atomic bonds.

Table S1 lists the values of the indexes for all the oxygen-hydrogen bonds in the water dimer. The values are normalized to the index obtained for the archetypal HB of the water dimer. The two-orders of magnitude that differentiate CB indexes from HB index are indicative of the higher electrical inertia of the CB when comparing with HB.

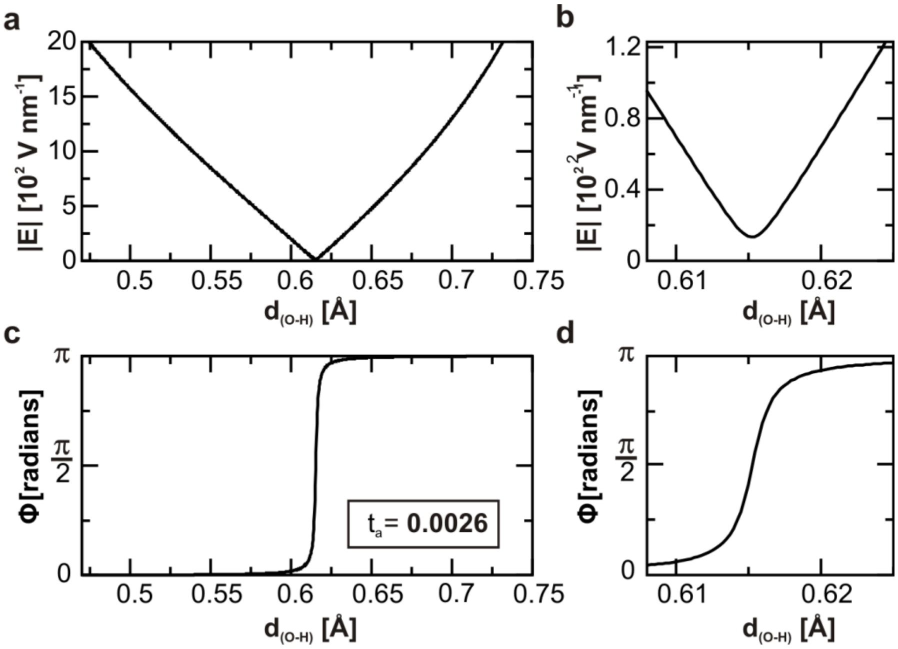

Figure S3. Electrical characterization of CB for the water dimer. a and **b,** Representation of the variation of the

electric field modulus (|**E**|) along the bond axis. The asymptotic behaviours coincide with O and H positions (positive charge centres in our model). **c** and **d**, Characterization of the evolution of the angle between **E** and the interaction axis, along the bond length, Φ= Φ(d). The deviations of such curves from the ideal Heaviside function are an indication of the electric susceptibility of chemical bonds. The descriptor included in the insets accounts for this information.

**Bond Label**	$t_a / t_a(O' \cdots H)$
**O-H$_{W1}$**	0.025
**O-H$_{W2}$**	0.024
**O-H$_{W3}$**	0.024
**O-H$_{W4}$**	0.025

Table S1. Numerical results for the electric field descriptor, t_a, describing O-H covalent bonds in the water dimer. Results are normalized to the HB descriptor in the dimer.

Fig. S4 is a summary of the analysis carried out to estimate errors in numerical results. Regarding the *ab-initio* models we highlight the imperceptible influence of the exchange model in the mean field potentials and the completeness of the basis set as shown in Figure´s S4a and S4c. This result agrees with the fulfilment of the Hellmann-Feynman theorem for electrostatic forces and its relation with the selection of the exchange-correlation potential within *ab-initio* models. In regards with basis sets selection we have applied an accepted reasoning in our field, we increase the basis sets size until we fix the error below a selected limit[11].

3. On the Electrical Nature of HB.

The dipole model for HB or polar covalent bonds is the most common approach for treating electrostatic interactions of these bonded systems under a classic (molecular mechanics) framework. However, the consideration of X-H bonds as dipoles is limited to effects occurring at

distances longer than the bond distance, otherwise such bonds must be studied under a many-bodies quantum mechanics framework.

Then, here we have introduced a novel electrical description of chemical interactions, using an improved DFT approach[11] and *ad-hoc* developments, Supp. Mat. 1 for further details. This approach permits to derive a mean-field structural (electron density) parameter that characterizes, similar to a classical fashion, the most native electrical features of chemical bonds. This method complements the classical (molecular mechanics) view of hydrogen bonds in terms of a dipole model.

3.1 Regarding the limitations of the dipole model for HB within the bond region.

Analysis of **E**-projections in the main axis of the bonds (Fig. 3 in the main text) show two subtle features to remark (i) the asymptotic behaviors in |**E**|, coincide with the two atom positions, i.e.

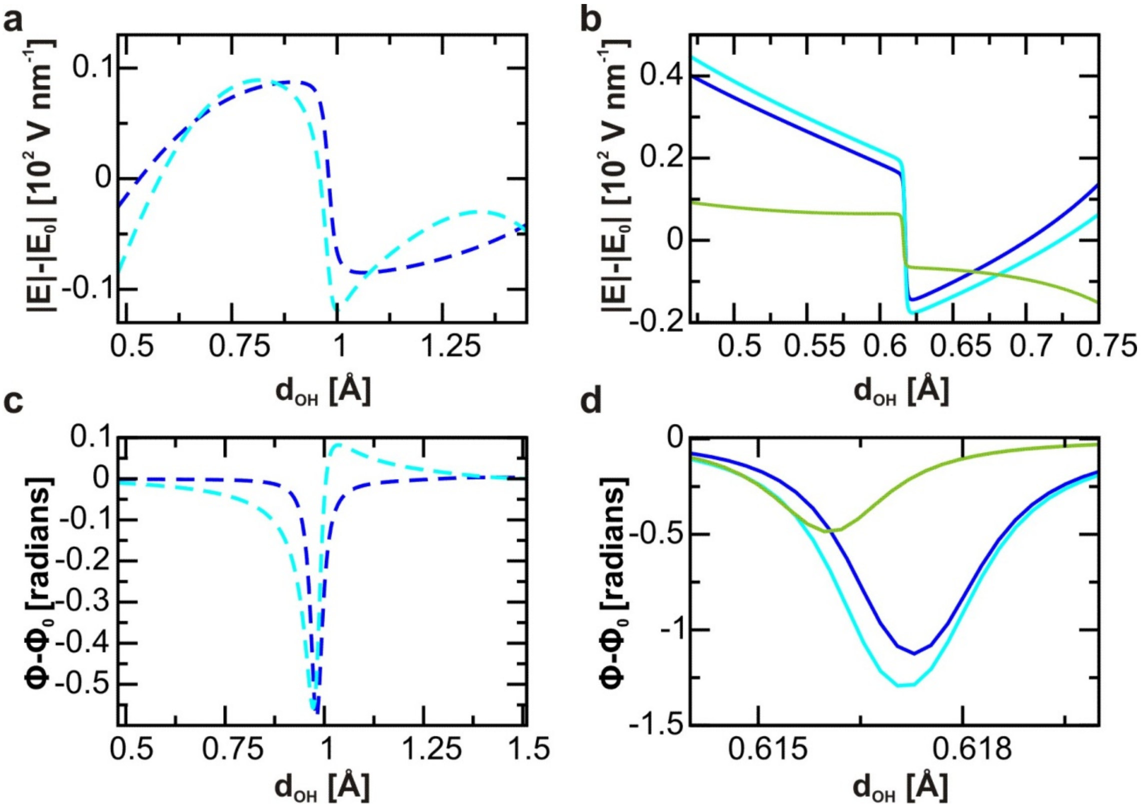

Figure S4. Estimation of the errors introduced by *ab-initio* methods. We report the deviation of the |**E**| and the Φ values relative to values reported in Figs. 2 and 3. The influence of mean-field potentials and as well as basis sets completeness for HB is represented in the panels **4a** and **4c**, and for the selected CB $(O'\text{-}H)_{W1}$ is indicated in **4b** and **4d**. In dark blue colour we represent the mean value deviations when the Fock exchange is 80 % of the total exchange. It represents an upper bar to the effect of the exchange in the model outcomes. As noticed in all the cases, the errors remain below 1%. By light blue colour we represent mean value deviations when 6-311g basis set is used. Relative errors remain below 10%. This higher value corresponds with the singular points, the poles in the eq. S2. Then, such points will not affect the numerical results which accounts for a limit in this equation.

charge centers, (ii) the dependence between the angle defined by **E** and the bond axis (Φ) (lower panels) shows that **E** undergoes 180º rotations. These observations constitute evidences of the inapplicability of a dipole model for a general HB within the interaction range. A dipole model must follow a constant relationship (Φ=0/π) in analytical representations as such included in

Fig.´s 3c, S4 (a) and (b), for Φ=Φ (d_{OH}). However, as could be seen from Fig. 3c the HB behavior seems closely related to a Heaviside Function, Fig. S5 (a). Deviations encountered in respect with this ideal behavior could be quantified by fitting multiple points (d_{OH}, $\Phi_{(d)}$) to a limiting Heaviside Function characterized giving rise to a finite t_a value (see also Supp. Mat. 1). Such behaviors numerically represented in Fig. 3 and interpreted via Fig.´s S5, support us to propose the interpretation of the electrostatic features of HB at interatomic distances in a picture of two interacting-positive-point charges modulated by surrounding electron clouds. It is precisely such polarized cloud the responsible for the deviations in respect with the ideal picture, Fig. S5 (a).

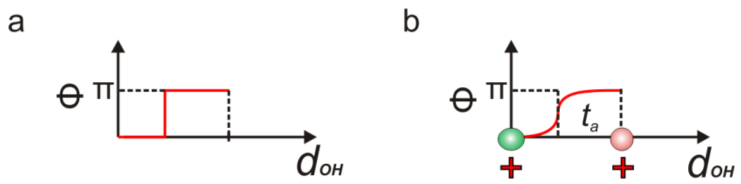

Figure S5. Electrostatic model for HB at intrabond scales. (a). Representation for a Φ=Φ (d_{OH}) responding to a Heaviside Function. **(b)** The deviation from such Heaviside function due to the existence of a finite electron density delocalized around the two positive charges. The parameter t_a quantifies such deviation.

3.2 About the extension of the dipole model to the comprehension of crystalline piezoelectric effects due to localized single-bonds piezoelectricity.

The PZ response through the entire crystal could be understood as a collective contribution of particular PZ effects localized in each bond. In the crystal scale, HB are the major contributors to PZ (red dotted lines in Fig.´s 1 and 2 in the main text). In such long-range reference, the electric effect (originated within the bond region of HB) could be modeled by induced variations in localized dipole moments (**p** in Fig. S6). Under this macroscopic view based for HB dipoles

($\mathbf{p_1}$...$\mathbf{p_6}$ in Fig. S6), the existence of a finite PZ effect is straightforward due to existence of finite resultant dipole moment. Considering crystalline symmetries, final contribution will be oriented as: $\mathbf{p_x} = \mathbf{p_{5x}} + \mathbf{p_{6x}}$ (interchains HB) and $\mathbf{p_y} = \mathbf{p_{1y}} + \mathbf{p_{2y}}$ (intrachain HB), see Fig. S6. Due to CB constrictions in the x-axis, we explain the atomic origin of the 2D-NC PZ response in terms of contributing $\mathbf{p_y}$, equivalently the dipole moments originated in intrachain HB.

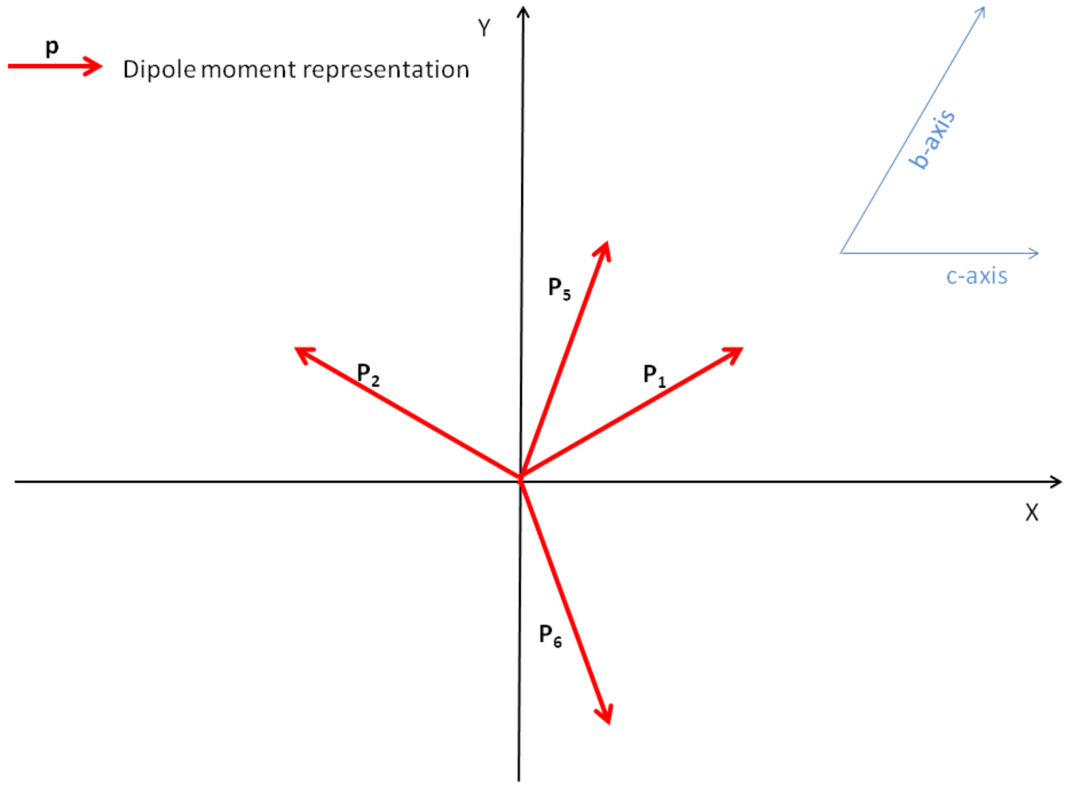

Figure S6. Representation of far-field HB dipole affecting PZ response in 2D-NC. $\mathbf{p_1}$, $\mathbf{p_2}$, $\mathbf{p_5}$, $\mathbf{p_6}$ represents dipole moments associated to O3-H…O5, O2-H…O6, O6-H…O3' and O6'-H'…O3 respectively.

4. Interpolation method for the definition of piezoelectric coefficients

Results reported in the main manuscript for the PZ coefficients correspond with the slopes of the linear interpolations done through a set of data (**E**-field, strain). The selection of a linear interpolation method corresponds with the accepted assumption[13] that we are dealing with a

linear response regime (mechanical relaxations in the nearby of the equilibrium position and **E** values close to electrical threshold).

Tables S2 summarizes the interpolation results for the behaviours represented in Fig. 5a.

Bond label\ Statistical Method	**Lineal** $\boldsymbol{\varepsilon_{22}}$ **± std. err. [pm V^{-1}] (Adj. R^2) [p-value] {intercept}**	**Polynomial** $\boldsymbol{\varepsilon_{22}}$ **± std. err. [pm V^{-1}] (adj. R^2) [p-value] {intercept}**
O_3-H···O_5	10.065 ± 1.426 (0.830) [0.000059] {0.0131}	10.065 ± 0.520 (0.977) [1x10^{-7}]
O_6-H···O_3'	4.278 ± 0.399 (0.919) [0.000002] {-0.0041}	4.278 ± 0.132 (0.992) [2x10^{-9}]
O_2-H···O_6	35.573 ± 3.863 (0.893) [0.000007] {0.0298}	35.573 ± 2.285 (0.963) [0.000001]

Table S2. PZ estimation at the level of localized single-bonds contribution. Statistical analysis for the data presented in Fig. 5a.

As could be distinguished, the linear coefficients (estimated PZ coefficient) of both models show the same mean value which is indicative of the robustness of the prediction and the validity of the linear regime. Although the quadratic model evidently shows a better adjustment, the linear approaches are all statistically significant (note that the models' p-values are inferior to 0.05) which also supports the linear framework.

REFERENCES

1. Arunan, E. *et. al.* Defining the hydrogen bond: An account (IUPAC Technical Report). *Pure and Applied Chemistry* **83**, 1619-1636 (2011).

2. Steiner, T. The Hydrogen Bond in the Solid State. *Angewandte Chemie-International Edition* **41**, 48-76 (2002).

3.Todeschini, R. and V. Consonni. Handbook of Molecular Descriptors, Wiley-VCH Verlag GmbH (2008)

4. Mishima O. & Stanley, H. E. *Nature* **1998**, 396, 329-335.

5. Muller-Dethlefs, K. & Hobza, P. *Chem. Rev.* **2000,** 100, 143-167 (2000).

6. Ludwig, R. *Angew. Chem. Int. Edit* **2001**, 40, 1808-1827.

7. Kohn, W. and Sham, L. J. *Phys. Rev.* **1965**, 140, 1133–1138.

8. Perdew, J. P. and Wang, Y. *Phys. Rev. B* **1986, 33**, 8800-8802.

9. Perdew, J. P. *et al*. *Phys. Rev. B* **1992, 46**, 6671-6687.

10. Staroverov, V. N. ,Scuseria, G. E. ,Tao, J. and Perdew, J. P. *J. Chem. Phys.* **2003,** 119, 12129-12137.

11. García, Y. & Sancho-García, J.C. *J. Chem. Phys.* **2008,** 129, 034702.

12. Sobczyk, L., Grabowski, SJ. & Krygowski, TM. *Chemical Reviews* **2005,** 105, 3513-3560.

13. Werling, K. A., Hutchison G. R. & Lambrecht, D. S. *Journal of Physical Chemistry Letters* **4**, 1365−1370 (2013).
